# Recentring young people in AI for mental health support: breaking the evidence-design feedback loop

**DOI:** 10.3389/fpubh.2026.1954336

**Published:** 2026-10-14

**Authors:** Jaspreet Kaur Sangha, Stephanie R. Partridge, Rebecca Raeside

**Affiliations:** 1 Faculty of Medicine, Imperial College London, London, United Kingdom; 2 Faculty of Medicine and Health, The University of Sydney, Sydney, NSW, Australia; 3 Charles Perkins Centre, The University of Sydney, Sydney, NSW, Australia

**Keywords:** adolescents, AI, artificial intelligence, co-design, health equity, mental health, young people

## Abstract

Young people are largely underrepresented in research underpinning AI-based mental health tools. AI systems are shaped by evidence bases that do not always fully incorporate young people’s opinions and perspectives. We propose a conceptual model, an evidence-design feedback loop in which exclusion from research may risk producing interventions that are less likely to be adopted by young people or meet their needs. These risks are amplified for young people from marginalised and underrepresented cultural contexts who face compounded exclusion, as the evidence base and design processes often lack cultural diversity. The relationships in this model are hypothesised from existing literature. This article argues that structural change is required across both research and industry to incorporate co-design with young people. AI developers should demonstrate meaningful involvement of young people from the earliest stages of development, and regulators should enforce higher regulatory standards in this respect. Researchers and AI developers should embed meaningful co-design with young people into the development of AI-based mental health tools which may help these technologies to be more relevant, safe and responsive to young people’s lived experiences.

## Introduction

AI-based mental health tools for young people are proliferating rapidly, with interventions such as chatbots increasingly positioned as scalable solutions to rising demand (1). The term ‘young people’ is used to refer to individuals aged 10–24 years, encompassing adolescents aged 10–19 and young adults aged 20–24. This reflects evidence that aspects of biological, psychological and social development continue throughout these age ranges (2). Where cited studies define age groups differently, age ranges are reported where relevant. AI refers to the range of subsets increasingly applied in this space, including machine learning, natural language processing, and generative AI, all of which are shaped by existing datasets and evidence to function and improve (3). These AI tools are often shaped by an evidence base that has largely excluded the young people they are designed to serve. This mismatch can risk producing interventions that are ineffective, unsafe, and inequitable (4).

There are three related but distinct concepts. Research participation refers to young people’s inclusion as subjects without necessarily having influence over study design (5). Engagement or involvement with young people refers to advisory input at various stages of study design usually without conferring decision-making power (5). Co-design refers to shared decision-making power between young people and adults throughout the processes of design and implementation. The argument presented here concerns the concept of co-design ensuring that young people have genuine influence over AI-based mental health tools built for them (6).

Consider a potential development pathway. Developers may frame the problem using existing clinical literature, make decisions around interface and key features without meaningful co-design, and draw on knowledge sources and evaluation criteria derived from studies that rarely engage young people. Where relevant, the tool may also be trained or fine-tuned on data reflecting the same evidence base. It may be that only after launch are users invited to provide feedback, once core design decisions are already fixed. This could then eventually lead to declining usage and limited sustained impact.

This is not simply a design problem. It reflects an important gap spanning both research and technology development. Young people remain markedly underrepresented in health research, with only a minority of studies incorporating their views (7). This may contribute to an evidence base that does not adequately reflect their lived experiences, preferences, or risks. This may consequently be reflected in how developers frame the problem the AI tool addresses, the design decisions they make, and the clinical, behavioral, evaluation and safety criteria they draw on. Where AI systems are trained or fine-tuned on data related to this evidence base, these limitations may also be reflected in their outputs. Whilst this exclusion does not solely impact young people, its consequences are often felt more strongly in this group especially for adolescents as their health-seeking behaviors and risk perceptions are in crucial developmental stages (8).

We propose that these relationships can be conceptualised as an evidence-design feedback loop in which exclusion at the research stage may lead to misaligned AI interventions, which in turn may reinforce low engagement and limited effectiveness. The relationships in this model are hypothesised and draw on existing literature. Without intervention, this hypothesised cycle risks entrenching inequities, particularly for young people from culturally diverse and marginalised backgrounds and may undermine the clinical potential of AI in mental health care.

Take Tessa as an example. Tessa was a chatbot from the NEDA (National Eating Disorders Association) aimed at supporting people with body image and eating disorders, a mental health issue more prevalent among adolescent girls and young women (9). After launching, Tessa was reportedly updated without prior consultation with NEDA to incorporate a new generative AI feature. It is not established whether the developer conducted testing with the target population before this update. Reports subsequently identified inappropriate advice Tessa was suggesting, such as calorie restrictions, avoiding “unhealthy foods,” and promoting diet culture (10). Such advice is inconsistent with best practice in eating disorder management (11). NEDA then indefinitely suspended the chatbot. This is particularly concerning given that adolescence is a peak period of onset for eating disorders (9). At a time when body image and self-worth can be more closely tied up with peer comparison (12), a chatbot reinforcing calorie restriction to a user in this developmental window risks entrenching harm in ways that generic safety systems may fail to detect, particularly when they do not account for adolescent vulnerabilities (13). Although Tessa was developed to support those with eating disorders specifically, this example highlights a wider concern; inappropriate or harmful outputs may be produced when young people’s perspectives and co-design are insufficiently incorporated into the development of AI health tools.

Conversely, the Grové study of an AI-powered mental health chatbot provides a concrete example of co-design in practice (14). Study of an AI-powered mental health chatbot provides a concrete example of co-design in practice. Researchers engaged a youth reference group with adolescents aged 15–17 from the earliest phase to inform chatbot design. They deliberately built rapport and incorporated recommendations from young participants immediately. This input resulted in the chatbot becoming gender neutral and a broader range of topics such as drugs, alcohol and sexuality being included as this is what young people reported as being relevant for them. Young people also directly shaped the chatbot’s linguistic style. Although this study illustrates meaningful co-design, similar examples remain rare (5).

### The problem begins before the tool

One in seven adolescents aged between 10 and 19 years old have a mental health disorder (15). Despite a high mental health burden, this population group expresses a reluctance to access external support with a preference for self-coping strategies (15). To address this issue, AI is now being integrated into mental health tools to build personalised interventions (16). Yet the rapid integration of AI into mental health tools has not been matched by meaningful co-design with young people.

The *Lancet Child & Adolescent Health* commission found that adolescents were involved in only approximately 15% of adolescent health research in a capacity beyond that of research participants (7). This strikingly low level of involvement reveals the overall extent of their exclusion in the field broadly. Poor integration of young people’s perspectives within broader health research translates downstream, limiting the inclusion of their voices within mental health specific research. A 2025 systematic review of 41 unique co-design studies with young people in the development of mental health interventions found that none of the included studies demonstrated a high degree of participation from young people in co-design, while more than 50% showed a low degree of participation (6). This limited involvement may contribute to an evidence base that does not accurately reflect young people’s lived experience, limiting the relevance of downstream AI interventions.

This gap is not contained within research; it is reproduced and amplified in AI-based mental health tool development. A recent review of 114 studies found that more than half of the included studies did not engage young people aged 10–25 years in app design and development (4). High-quality co-design ensures shared decision-making power between young people and adults and gives equal value to lived experience and professional expertise (17). However, currently even when researchers do engage young people in co-design processes, the quality is not always high, and engagement levels often remain low.

### How AI could amplify the exclusion

AI-based mental health tools draw on existing evidence bases and training datasets. Therefore any biases, exclusions, or knowledge gaps present within these sources may be perpetuated and amplified through the interventions they generate (1). This evidence base comes from research that already underrepresents or excludes young people’s perspectives, particularly those from historically marginalised backgrounds (6, 16). It informs how developers formulate the problem, make design decisions and set evaluation and safety criteria, drawing on sources such as existing clinical literature and outcome measures. Where these gaps exist, they may therefore be carried into the resulting tools. Taken together, these relationships suggest a potential self-reinforcing feedback loop, as Figure 1 conceptualises, in which an evidence base built without young people shapes the development of AI-based tools that are then delivered to them. This proposed cycle may increase the likelihood that AI health interventions will fail to engage young people or meet their needs.

**Figure 1:**
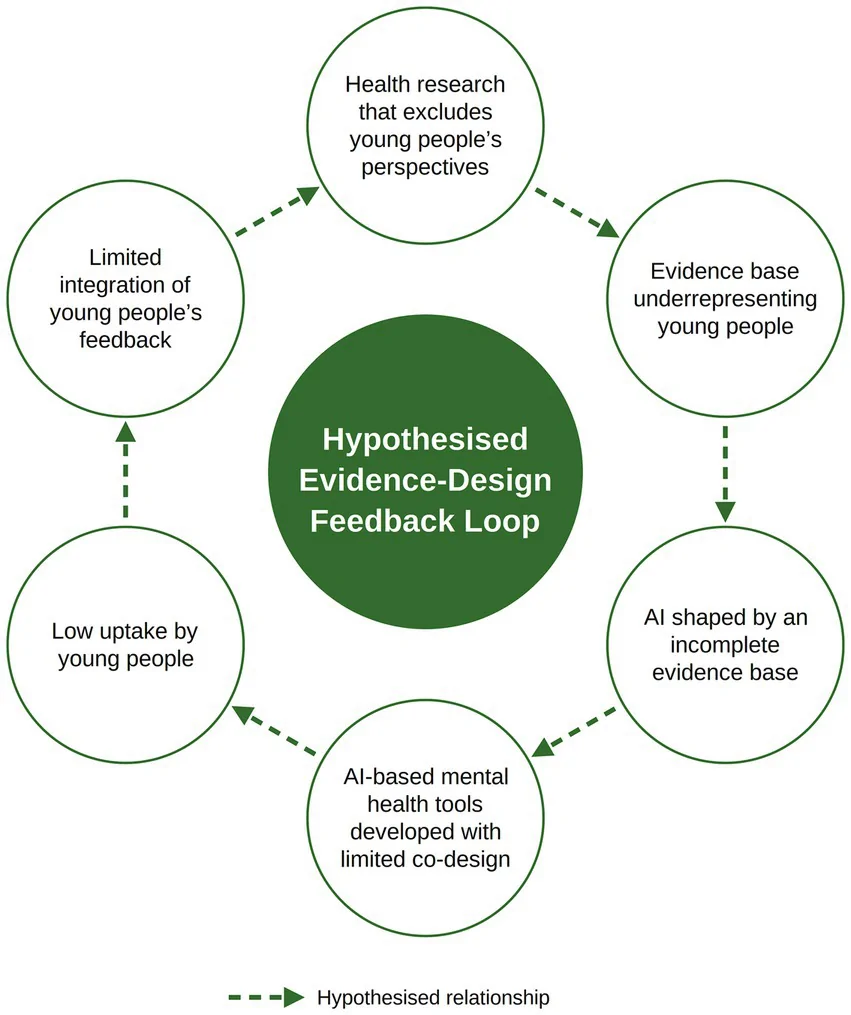
A proposed exclusion feedback loop in AI-based mental health tool development for young people. Health research that largely excludes young people generates an evidence base that underrepresents them. AI shaped by this incomplete evidence base may be used to help develop mental health apps that are not co-designed with young people, which may contribute to lower uptake and safety risks. This in turn may contribute to continued exclusion from the next generation of research and development. Arrows indicate hypothesised relationships.

### Risks specific to young people

The case for co-design with young people extends beyond increasing uptake of interventions. Young people have distinct expectations regarding online security, autonomy, and digital interaction, and ignoring these expectations could introduce risks, which may include ineffective therapeutic support, misinterpretation of distress signals, and reduced help-seeking behavior (18, 19). Young people face a specific set of risks when interacting with AI, although some developmental vulnerabilities are particularly relevant during adolescence. Two developmental features help to explain why. First, adolescent egocentrism heightens adolescents’ sense of being watched and judged by others which is termed the imaginary audience and personal fable (20). This can make them particularly vulnerable to AI content encouraging comparison, with risks such as the development of harmful body image standards or encouragement of self-harm intentions, as a recent report has demonstrated (21). Second, the dual systems model proposes that the socioemotional system, which drives sensitivity to reward, matures earlier than the cognitive control system responsible for self-regulation, so adolescents may be more responsive to potential rewards than to potential risks (8). This may mean they do not reliably discount potentially harmful AI advice presented to them (8). Together, these developmental features can mean that adolescents sometimes struggle to assess risks to themselves depending on their age and developmental trajectory (2).

Including young people in the co-design process is vital as they hold first-hand insight into what does and does not work for them, as well as an awareness of stressors that they experience which existing research may not fully capture. However, co-design is one component of a broader safety framework and is not sufficient alone to ensure the development of appropriate AI-based mental health tools. Co-design must not shift responsibility solely onto young people and must be balanced with clinical experience and expertise to account for the developmental stages of adolescence and young adulthood and the vulnerabilities it may come with. A comprehensive approach would also include appropriate clinical evaluation and safety testing in realistic adolescent crisis scenarios before deployment as well as human oversight with clear escalation procedures for users at risk, and ongoing monitoring after release (22). The Lundy model can help strike a balance between co-design and appropriate risk management. The model describes the need to include an audience of suitable professionals who are listening to and able to action the young people’s opinions, allowing harm to be mitigated appropriately (23). Ensuring young people’s safety when they use AI-based mental health tools is vital to achieving therapeutic goals without causing harm.

### The particular failure for underrepresented cultural contexts

Cultural relevance plays an essential role in ensuring that AI-based mental health tools deliver appropriate therapy. Currently, minority groups of young people may experience a particularly strong lack of this relevance as the existing data represent these groups even less. AI-based tools shaped by this evidence base, through problem formulation, design decisions, evaluation criteria and, where relevant, training data, may therefore lack the cultural competence and nuance needed to deliver appropriate therapies or strategies (24).

For example, a study from India showed that Indian adolescents felt that existing AI chatbots lacked cultural relevance and sufficient personalisation (25). Another mixed-methods study highlighted broader cultural differences, showing that chatbot users from Canada, the UK and USA tend to express themselves more directly and seek solutions, while those from Indonesia, India, Malaysia, the Philippines and Thailand adopt more implicit communication styles and prefer solutions that promote family harmony (26). The study did not record users’ ages, although young adults were reported to be the most common users.

Algorithmic bias is an important digital determinant of health and several biases, including racial and gender-based biases, have already been identified within AI algorithms (27). For AI-based mental health tools to serve young people effectively, they must respond to each young person’s background and context. Co-design with young people from diverse backgrounds in creating these tools could help identify and address culturally inappropriate or biased support strategies.

### Challenges

Barriers to co-design with young people in this context are real and require direct acknowledgement. Lengthy and complex ethical approval processes for research involving young people can discourage engagement (28). Furthermore, ethical review boards themselves are attempting to navigate the use and ethics of AI and research (29). Researchers also do not always allocate appropriate reimbursement for young people’s time within research budgets (17). Navigating power dynamics can additionally introduce challenges in co-design and development processes (17). Other challenges include difficulties with selection bias resulting in unrepresentative samples, tokenistic involvement which does not reflect true co-design, and continued exclusion of marginalised groups of young people who face barriers to meaningful co-design within existing infrastructure (30).

However, these barriers should not prevent young people from participating in developing interventions that ultimately affect them. The UN Convention on the Rights of the Child identifies respect for children’s views as a fundamental right (31). Resource constraints and logistical difficulties do not automatically ensure that these rights are upheld, but they should not override this principle. Stakeholders must actively address these challenges to ensure that they include young people’s perspectives when developing AI-based mental health tools.

One way to mitigate some of this complexity involves using UNICEF’s guidance on children and AI governance, which supports the development and evaluation of AI guidelines and policies (32). This guidance states that young people must have agency and every opportunity to shape AI systems, supporting their involvement in developing AI-based mental health tools. Various countries are now also developing guidance for the use of AI in healthcare. Australia has published information for both patients and professionals to describe safe use of AI and regulation of AI tools in healthcare (33). Similarly, the UK has recently concluded a call for evidence on the use of AI in healthcare as part of its National Commission into the Regulation of AI in Healthcare (34). Structured case studies are also available with Chang et al. describing an approach for supporting young people as co-designers of AI systems, particularly among historically marginalised groups, by embedding ethical commitments throughout the process (35).

## Discussion

Because the problem is structural, incremental adjustments will be insufficient; coordinated changes across research and regulation are required. Meaningful change must occur at two levels simultaneously: within the research that underpins these tools and within the industry governance that regulates them.

Researchers bear the first responsibility. They must build a more representative evidence base that includes young people’s perspectives directly with a greater number and range of young people being included from different backgrounds. Young people must not be treated as a homogenous category during recruitment and reporting of data to help distinguish the varied levels of development within adolescence and young adulthood. Outcome measures that are specifically validated for use in young people should also be included (36). Funding bodies and institutions should require demonstrated involvement of young people as an expectation within research funding criteria and institutional research governance, where appropriate to the study design. Existing frameworks, such as the Youth Engagement in Research Framework from INGUAGE, already provide guidance on genuine co-design with young people rather than tokenistic consultation (37). Embedding such frameworks into grant requirements and publication criteria could help address the proposed feedback loop at its source, supporting researchers to build the evidence base with young people rather than about them.

Researchers must also routinely allocate funding to reimburse young people appropriately for their time. Treating participation as a funded activity, rather than a charitable voluntary contribution, represents an important ethical consideration for sustained engagement (38).

AI developers share equal responsibility, although current regulatory environments do not yet enforce it. AI developers may not always have the expertise or structures required for meaningful co-design with young people, and commercial incentives may not always align with the interests of users (39). Regulators must therefore hold these companies to a higher standard. Developers should demonstrate meaningful involvement of young people from the beginning of the development process, rather than limiting engagement to retrospective testing.

Another way to recentre young people in the development of AI for mental health support is to incorporate developmental theory. This can support translating input from co-design with young people into concrete design principles. For example, the dual systems model highlights how heightened reward sensitivity and still-developing self-regulation shapes risk-taking in adolescents, and so can be used to develop risk identification models and escalation protocols specific to this group that account for developmental characteristics (8). Additionally, imaginary audience as part of the adolescent egocentrism theory can be used to guide design and language choices for adolescents that avoid amplifying self-consciousness and provide support in key mental health areas such as perceived scrutiny and judgement (20).

UNICEF guidance can support this process and serve as a foundation for standardised methods and frameworks (32). Crucially, developers should be expected to demonstrate that their co-design process is comprehensive and inclusive of young people from diverse backgrounds and ages. Incorporating these criteria into market authorisation processes for particularly high-risk AI mental health tools would help to improve transparency and ensure cultural relevance for all users. Stronger regulatory oversight and clearly defined standards are necessary to ensure that developers incorporate young people’s perspectives early in the design process, with the level of scrutiny for market authorisation proportionate to the potential risks associated with the tool.

Neither approach alone is likely to succeed. Research that includes young people will remain ineffective unless industry acts on it. Conversely, regulation that mandates involvement of young people will lack substance without a representative evidence base. Both must advance together.

Without intervention, the rapid integration of AI into mental health care risks embedding longstanding exclusions into new technological systems at scale. The tools to help address this already exist. Embedding meaningful co-design with young people, particularly with those from underrepresented backgrounds, across research and development could improve engagement with AI-based mental health tools. Importantly, this could also improve the relevance, and equity of such tools (4). The question is no longer whether young people should be involved, but whether stakeholders are willing to redesign systems to ensure that they are.

## Data Availability

The original contributions presented in the study are included in the article/supplementary material, further inquiries can be directed to the corresponding author.

## References

[1] Matthews E Cleary F Firth J. Considerations about the proliferation of large language model chatbots and youth mental health. Ir J Psychol Med. (2026) (First View)):1–4. doi: 10.1017/ipm.2026.10195 42027103

[2] Sawyer SM Azzopardi PS Wickremarathne D Patton GC. The age of adolescence. Lancet Child Adolesc Health. (2018) 2:223–8. doi: 10.1016/S2352-4642(18)30022-1, 30169257

[3] Yu P Xu H Hu X Deng C. Leveraging generative artificial intelligence and large language models: a comprehensive roadmap for healthcare integration. Healthcare (Basel). (2023) 11:2776. doi: 10.3390/healthcare11202776 PMC10606429 37893850

[4] Figueroa C Pérez-Flores NJ Guan KW Zhang R Orchard N Kuebler W . Designing for all youth: a scoping review of equity and participation in mental health apps. NPJ Digit Public Health. (2026) 1:8. doi: 10.1038/s44482-026-00012-y

[5] Sanchez S Thorburn R Rea M Kaufman P Schwartz R Selby P . A systematic review of theories, models and frameworks used for youth engagement in health research. Health Expect. (2024) 27:e13975. doi: 10.1111/hex.13975, 39102670 PMC10825621

[6] Chinsen A. Berg A. Nielsen S. Trewella K. Cronin T. J. Pace C. C., et al. Co-design methodologies to develop mental health interventions with young people: a systematic review. Lancet Child Adolesc Health (2025) 9:413–425. doi: 10.1016/S2352-4642(25)00063-X 40311649

[7] Nagata JM Imbago-Jácome D Choonara S Talebloo J Memon Z O’Sullivan M . Reporting of research with adolescent and youth engagement. Lancet Child Adolesc Health. (2025) 9:442–5. doi: 10.1016/S2352-4642(25)00092-6 40409336

[8] Steinberg L. A dual systems model of adolescent risk-taking. Dev Psychobiol. (2010) 52:216–24. doi: 10.1002/dev.20445, 20213754

[9] López-Gil JF García-Hermoso A Smith L Firth J Trott M Mesas AE . Global proportion of disordered eating in children and adolescents: a systematic review and meta-analysis. JAMA Pediatr. (2023) 177:363–72. doi: 10.1001/jamapediatrics.2022.5848, 36806880 PMC9941974

[10] Wells K. An eating disorders chatbot offered dieting advice, raising fears about ai in health (2023). Available online at: https://www.npr.org/sections/health-shots/2023/06/08/1180838096/an-eating-disorders-chatbot-offered-dieting-advice-raising-fears-about-ai-in-hea (accessed May 20, 2026).

[11] American Psychiatric Association. The American Psychiatric Association Practice Guideline for the Treatment of Patients with Eating Disorders. 4th ed. Washington, DC: American Psychiatric Association Publishing (2023).

[12] Hamel AE Zaitsoff SL Taylor A Menna R Grange DL. Body-related social comparison and disordered eating among adolescent females with an eating disorder, depressive disorder, and healthy controls. Nutrients. (2012) 4:1260–72. doi: 10.3390/nu4091260, 23112914 PMC3475236

[13] Giovanelli A Roundfield KD. Adolescent vulnerability to consumer chatbots—artificial agents and genuine risk. JAMA Netw Open. (2025) 8:e2539028. doi: 10.1001/jamanetworkopen.2025.39028, 41129156

[14] Grové C. Co-developing a mental health and wellbeing chatbot with and for young people. Front Psych. (2021) 11:606041. doi: 10.3389/fpsyt.2020.606041, 33597898 PMC7882508

[15] World Health Organization. Mental health of adolescents (2025). Available online at: https://www.who.int/news-room/fact-sheets/detail/adolescent-mental-health (accessed May 23, 2026)

[16] Balan R Dobrean A Poetar CR. Use of automated conversational agents in improving young population mental health: a scoping review. NPJ Digit Med. (2024) 7:75. doi: 10.1038/s41746-024-01072-1, 38503909 PMC10951258

[17] Digital Transformations for Health Lab Shaping AI Governance for young People: Perspectives on Health, Policy and Regulation Geneva Digital Transformations for Health Lab (2026). Available online at: https://www.dthlab.org/articles/shaping-ai-governance-for-young-people (Accessed July 28, 2026).

[18] Ferretti A Hubbs S Vayena E. Global youth perspectives on digital health promotion: a scoping review. BMC Digit Health. (2023) 1:1–12. doi: 10.1186/s44247-023-00025-0, 38164791

[19] Schleider JL Morris R. Debate: conversational AI and young people's mental health—friend or foe? A case for designing conversational AI as a bridge to youth mental health support. Child Adolesc Ment Health. (2026) 31:229–31. doi: 10.1111/camh.70105, 42389998 PMC13474295

[20] Popovac M Hadlington L. Exploring the role of egocentrism and fear of missing out on online risk behaviours among adolescents in South Africa. Int J Adolesc Youth. (2020) 25:276–91. doi: 10.1080/02673843.2019.1617171

[21] Yousif N. Parents of teenager who took his own life sue OpenAI (2025). Available online at: https://www.bbc.com/news/articles/cgerwp7rdlvo (accessed May 21, 2026).

[22] Olisaeloka L Richardson CG Wang AY Munthali RJ Vigo DV. Safety mechanisms and risk mitigation in generative AI mental health chatbots: a systematic scoping review. Healthcare. (2026) 14:1395. doi: 10.3390/healthcare14101395, 42194487 PMC13205439

[23] Child Outcomes Research Consortium (CORC). The Lundy model of participation (n.d.). Available online at: https://www.corc.uk.net/for-young-people/the-lundy-model-of-participation/ (accessed August 27, 2026)

[24] Azzopardi P Clark TC Renfrew L Habito M Ameratunga S. Advancing impactful research for adolescent health and wellbeing: key principles and required technical investments. J Adolesc Health. (2024) 75:S47–61. doi: 10.1016/j.jadohealth.2024.04.001, 39293877

[25] Sehgal NKR Kambhamettu H Matam SP Ungar L Guntuku SC Exploring socio-cultural challenges and opportunities in designing mental health chatbots for adolescents in India. Proceedings of the extended abstracts of the chi conference on human factors in computing systems New York, NY, USA ACM (2025) 1–7

[26] Chin H Song H Baek G Shin M Jung C Cha M . The potential of chatbots for emotional support and promoting mental well-being in different cultures: mixed methods study. J Med Internet Res. (2023) 25:e51712. doi: 10.2196/51712, 37862063 PMC10625083

[27] Chidambaram S Jain B Jain U Mwavu R Baru R Thomas B . An introduction to digital determinants of health. PLOS Digit Health. (2024) 3:e0000346. doi: 10.1371/journal.pdig.0000346, 38175828 PMC10766177

[28] Warraitch A Lee M Bruce D Curran P Khraisha Q Wacker C . An umbrella review of reviews on challenges to meaningful adolescent involvement in health research. Health Expect. (2024) 27:e13980. doi: 10.1111/hex.13980, 39102665 PMC10821743

[29] National Health and Medical Research Council Guide for assessing research involving artificial intelligence, machine learning and large language model technology (collectively “AI”) (2025). Available online at: https://www.nhmrc.gov.au/about-us/resources/guide-assessing-research-involving-artificial-intelligence-machine-learning-and-large-language-model-technology-collectively-ai (accessed May 19, 2026)

[30] Shahnaz I Rose-Clarke K Michelson D Gronholm PC. Codesign of mental health interventions with young people from racially minoritised populations: a systematic review of methods and outcomes. Health Expect. (2025) 28:e70204. doi: 10.1111/hex.70204, 40047285 PMC11883660

[31] United Nations Convention on the rights of the child text (1989). Available online at: https://www.unicef.org/child-rights-convention/convention-text (accessed May 20, 2026).

[32] UNICEF Innocenti—Global office of Research and Foresight UNICEF Guidance on AI and Children 3.0 Florence UNICEF Innocenti (2025). Available online at: https://www.unicef.org/innocenti/media/11991/file/UNICEF-Innocenti-Guidance-on-AI-and-Children-3-2025.pdf (Accessed May 20, 2026).

[33] Department of Health Disability and Ageing Artificial Intelligence (AI) in health care (2026). Available online at: https://www.health.gov.au/topics/health-technologies-and-digital-health/what-we-do/artificial-intelligence-ai-in-health-care?language=en (accessed May 25, 2026)

[34] Medicines and Healthcare products regulatory agency. Closed call for evidence regulation of AI in healthcare (2025). Available online at: https://www.gov.uk/government/calls-for-evidence/regulation-of-ai-in-healthcare (accessed May 26, 2026)

[35] Chang MA Tissenbaum M Philip TM D’Mello SK. Co-designing AI with youth partners: enabling ideal classroom relationships through a novel AI relational privacy ethical framework. Comput Educ Artif Intell. (2025) 8:100364. doi: 10.1016/j.caeai.2025.100364

[36] Kwan B Rickwood DJ. A systematic review of mental health outcome measures for young people aged 12 to 25 years. BMC Psychiatry. (2015) 15:279. doi: 10.1186/s12888-015-0664-x, 26573269 PMC4647516

[37] Woodgate R. L. Youth engagement in research framework (n.d.). Available online at: https://www.ingauge.ca/researchers/#framework-youth (accessed May 20, 2026)

[38] Tisdall EKM Robinson C Battaglia SM Shukul S Ruiz-Casares M D'souza NA. Reframing payment practices for co-research for children and young people. Res Ethics. (2025) 22:322–34. doi: 10.1177/17470161251380888

[39] Blease C Tibbs M Balaskas A Liverpool S Hagström J Fitzgerald A. Coproduction without youth? Closing the participation gap in digital mental health research. JMIR Ment Health. (2026) 13:e91739. doi: 10.2196/91739, 42342243 PMC13347077

